# Humoral and cellular immune response to second and third severe acute respiratory syndrome coronavirus 2 mRNA vaccine in patients with plasma cell dyscrasia

**DOI:** 10.1002/cam4.5996

**Published:** 2023-04-26

**Authors:** Tomotaka Suzuki, Shigeru Kusumoto, Yoshiko Kamezaki, Hiroya Hashimoto, Nozomi Nishitarumizu, Yoko Nakanishi, Yukiyasu Kato, Akimi Kawai, Naohiro Matsunaga, Toru Ebina, Tomoyuki Nakamura, Yoshiaki Marumo, Kana Oiwa, Shiori Kinoshita, Tomoko Narita, Asahi Ito, Atsushi Inagaki, Masaki Ri, Hirokazu Komatsu, Takashi Aritsu, Shinsuke Iida

**Affiliations:** ^1^ Department of Hematology and Oncology Nagoya City University Graduate School of Medical Sciences Nagoya Japan; ^2^ Scientific Affairs Sysmex Corporation Kobe Japan; ^3^ Clinical Research Management Center Nagoya City University Hospital Nagoya Japan

**Keywords:** humoral and cellular immune response, mRNA vaccination, multiple myeloma, plasma cell dyscrasia, SARS‐CoV‐2

## Abstract

**Background:**

The recently developed severe acute respiratory syndrome coronavirus 2 (SARS‐CoV‐2) mRNA vaccine has a short history of use and further information is needed regarding its efficacy, especially in immunocompromised conditions, such as plasma cell dyscrasia (PCD).

**Methods:**

We retrospectively measured serum SARS‐CoV‐2 antibodies against the spike protein (S‐IgG) after the second and third mRNA vaccine doses (doses 2 and 3, respectively) in 109 patients with PCD. We evaluated the proportion of patients with an adequate humoral response (defined as S‐IgG titers ≥300 antibody units/mL).

**Results:**

Although active anti‐myeloma treatments prior to vaccination had a significantly negative impact on adequate humoral response, specific drug subclasses including immunomodulatory drugs, proteasome inhibitors, and monoclonal antibodies were not negatively associated, except for B‐cell maturation antigen‐targeted therapy. Dose 3 (booster vaccination) led to significantly higher S‐IgG titers and more patients acquired an adequate humoral response. Furthermore, evaluation of vaccine‐induced cellular immune response in patients using T‐spot Discovery SARS‐CoV‐2 kit, revealed an enhanced cellular immune response after Dose 3.

**Conclusions:**

This study highlighted the significance of booster SARS‐CoV‐2 mRNA vaccination in patients with PCD with respect to humoral and cellular immunity. Moreover, this study highlighted the potential impact of certain drug subclasses on vaccine‐induced humoral immune response.

## Graphical abstract

The current study demonstrated the significance of booster SARS‐CoV‐2 mRNA vaccination in patients with plasma cell dyscrasia with respect to humoral and cellular immunity. In addition, this study highlights the potential impact of certain drug subclasses on vaccine‐induced humoral immune response.

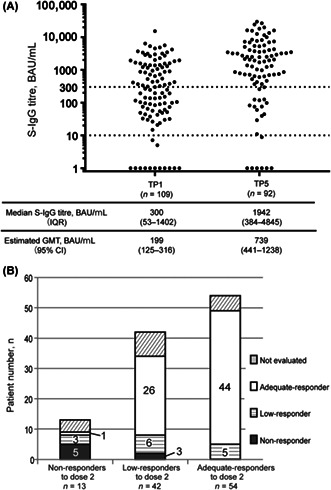

## 1 INTRODUCTION

The coronavirus disease (COVID‐19) pandemic caused by the severe acute respiratory syndrome coronavirus 2 (SARS‐CoV‐2) has severely affected patients with hematological malignancies, including plasma cell dyscrasia (PCD), leading to dismal clinical outcomes in these patients.^1^, ^2^, ^3^, ^4^ Data from the early stages of the pandemic revealed that patients with PCD who were hospitalized due to COVID‐19 demonstrated significantly higher mortality (~20%–30%)^1^, ^4^, ^5^ than age‐ and gender‐matched patients without cancer. Therefore, it is extremely important to prevent COVID‐19 infection and reduce the risk of developing severe COVID‐19 in patients with PCD, and the novel mRNA vaccines against SARS‐CoV‐2 were expected to be effective tools against COVID‐19 infection.^6^, ^7^

However, evaluation of SARS‐CoV‐2 antibody levels against the spike proteins (S‐IgG) in patients with PCD revealed a suboptimal immune response after two doses of mRNA vaccines.^8^, ^9^, ^10^, ^11^ Several studies have evaluated factors associated with humoral immune response after SARS‐CoV‐2 vaccination, and reported a negative association of pre‐vaccination active anti‐myeloma treatment with an adequate immune response.^8^, ^9^, ^10^ However, the effect of specific treatment subclasses on induced immune responses remained inadequately explored.^8^, ^9^, ^11^ Little information is available on induced cellular immune responses after mRNA vaccination.^12^, ^13^ Furthermore, a third vaccine dose is reported to induce a booster effect in patients with PCD^14^, ^15^; however, there is insufficient evidence of this effect. Thus, further research is required to understand the clinical efficacy of the mRNA vaccines.

In this retrospective observational study, we evaluated the vaccine‐induced humoral immune response by measuring S‐IgG titers after the second and third mRNA vaccine doses (Doses 2 and 3, respectively) in patients with PCD. Additionally, we evaluated the cellular immune response in a subset of patients. This study demonstrated the significance of booster SARS‐CoV‐2 mRNA vaccination in patients with PCD with respect to humoral and cellular immunity. In addition, this study highlights the potential impact of certain drug subclasses on vaccine‐induced humoral immune response.

## 2 MATERIALS AND METHODS

Eligible patients with PCD included those undergoing either active treatment or regular medical check‐ups at the Nagoya City University Hospital (NCUH), and who received at least two doses of the SARS‐CoV‐2 mRNA vaccine (BNT162b2 or mRNA‐1273). Other inclusion criteria were: (1) known vaccine type and time of mRNA vaccination and (2) available for stored serum sample collection between 7 and 60 days, defined as timepoint (TP) 1, after dose 2. Serum samples were collected between 91 and 120 days (TP2), 121 and 150 days (TP3), and 151 days or later (TP4) after dose 2. Furthermore, serum samples between 7 and 60 days (TP5) after dose 3 were collected (Figure S1).

### 2.1 Evaluation of humoral and cellular immune response to mRNA vaccination

S‐IgG and SARS‐CoV‐2 IgG antibodies against nucleocapsid (N) proteins (N‐IgG) were measured at Sysmex Scientific Affairs Laboratories, using a highly quantitative and reproducible assay, the HISCL® system (Sysmex Corp.), as previously described.^16^, ^17^

Cryopreserved peripheral blood mononuclear cells (PBMC) from eligible patients, obtained at TP1 and TP5 were used for the T‐spot Discovery SARS‐CoV‐2 assay (T‐SPOT assay) (Oxford Immunotec Ltd.) to evaluate vaccine‐induced cellular immune response. Briefly, cryopreserved PBMC samples were carefully thawed and only samples with a total cell count >1.0 × 10^6^ after thawing were used for the T‐SPOT assay. A total of 250,000 cells per well were conditioned and plated into individual wells of (1) SARS‐CoV‐2 spike antigens, (2) SARS‐CoV‐2 nucleocapsid antigens, (3) negative‐, and (4) positive controls. Cells were incubated and interferon‐γ (IFN‐γ)‐secreting T cells were detected. Spot‐forming units (SFU) were evaluated and patients with ≥10 SFU in spike antigen wells were determined as cellular responders.^13^, ^18^ For more information about humoral and cellular immune response evaluation, refer to the Supplementary Methods.

### 2.2 Analysis and ethical consideration

More information on statistical analysis is presented in the Supplementary Methods. According to the S‐IgG titers at TP1 and TP5, patients were classified as non‐responders (≤10 binding antibody units [BAU]/mL), low‐responders (>10 and <300 BAU/mL), and adequate‐responders (≥300 BAU/mL) to doses 2 and 3 as previously reported.^19^, ^20^, ^21^ Seroconversion was defined as acquiring an S‐IgG titer >10 BAU/mL.^19^, ^21^ The primary endpoint was the proportion of non‐responders, low‐responders, and adequate‐responders after doses 2 and 3, respectively. The secondary endpoint was clinical parameters associated with adequate‐responders after dose 2. In addition, serial changes in S‐IgG titer over time were evaluated using samples obtained at TP1 up to TP4. The S‐IgG titers and SFU in spike antigens wells of patients, whose cellular immune response was evaluated, were combined and evaluated for correlation.

This study was approved by the institutional review board of NCUH. Written informed consent was obtained from all participants to store their blood samples. This study was conducted in accordance with the Declaration of Helsinki.

## 3 RESULTS

A total of 109 patients with PCD were eligible for this study. The patient flow diagram is shown in Figure S2. Patients in this cohort received the first vaccine dose between May 28, 2021 and April 2, 2022. The clinical characteristics of the eligible patients are summarized in Table 1. The median age at dose 2 was 70 years (interquartile range [IQR], 63–77). The major underlying PCD was multiple myeloma (*n* = 104, 95.4%). The number of treatment lines prior to dose 2 was 1 (41.3%), 2 (22.9%), and 3 or more (32.1%), with median = 2. Eighty (73.4%) patients were undergoing active treatment, defined as any anti‐myeloma treatment within 3 months before vaccination.

**TABLE 1:** Patient characteristics at second mRNA vaccine dose (dose 2).

Factor	Overall	Non/low‐responders	Adequate‐responders	*p*
	*N* = 109	*n* = 55	*n* = 54	
Age at dose 2, years [IQR]	70 [63–77]	72 [69–77]	68 [60–75]	0.015
70 Years old or more, *n* (%)	61 (56.0)	36 (65.5)	25 (46.3)	0.055
Gender, male:female, *n*	60:49	29:26	31:23	0.701
Vaccine type, BNT162b2:mRNA‐1273, *n*	97:12	54:1	43:11	0.002
Disease				
MM, *n* (%)	104 (95.4)	52 (94.5)	52 (96.3)	0.618
AL amyloidosis, *n* (%)	3 (2.8)	1 (1.8)	2 (3.7)	
Other plasma cell dyscrasia, *n* (%)	2 (1.8)	2 (3.6)	0 (0.0)	
Involved immunoglobulin				
IgG, *n* (%)	62 (56.9)	30 (54.5)	32 (59.3)	0.517
IgA, *n* (%)	17 (15.6)	10 (18.2)	7 (13.0)	
BJP, *n* (%)	28 (25.7)	13 (23.6)	15 (27.8)	
Other, *n* (%)	2 (1.8)	2 (3.6)	0 (0.0)	
Number of lines of treatment, median, [IQR]	2 [1–3]	2 [1–3]	1.5 [1–2]	0.120
Before treatment, *n* (%)	4 (3.7)	2 (3.6)	2 (3.7)	0.359
1, *n* (%)	45 (41.3)	20 (36.4)	25 (46.3)	
2, *n* (%)	25 (22.9)	11 (20.0)	14 (25.9)	
3 or more, *n* (%)	35 (32.1)	22 (40.0)	13 (24.1)	
Received any anti‐myeloma treatments within 3 months before dose 2, *n* (%)	80 (73.4)	48 (87.3)	32 (59.3)	0.001
Prior ASCT, *n* (%)	45 (41.3)	15 (27.8)	30 (55.6)	0.004
Response at dose 2				
Before treatment/SD/PD, *n* (%)	29 (26.6)	16 (29.1)	13 (24.1)	0.261
PR, *n* (%)	28 (25.7)	17 (30.9)	11 (20.4)	
VGPR or more, *n* (%)	52 (47.7)	22 (40.0)	30 (55.6)	
Median lymphocyte count/μL, [IQR] (*n* = 105)	1311 [912–1872]	1325 [960–1935]	1286 [912–1638]	0.571
Lymphocyte count < 1000/μL, *n* (%)	32 (30.5)	14 (26.4)	18 (34.6)	0.402
Median albumin, g/dL, [IQR] (*n* = 105)	4.0 [3.7–4.3]	3.8 [3.6–4.1]	4.1 [3.9–4.4]	0.001
Albumin < 3.5 g/dL, *n* (%)	11 (10.5)	9 (17.0)	2 (3.8)	0.052
Median eGFR, mL/min/1.73 m^2^, [IQR] (*n* = 105)	62 [47–72]	58 [46–70]	64 [48–73]	0.238
eGFR < 40 mL/min/1.73 m^2^, *n* (%)	15 (14.3)	11 (20.8)	4 (7.7)	0.092
Median serum IgG level mg/dL, [IQR] (*n* = 103)	644 [373–1080]	499 [364–953]	806 [467–1100]	0.071
Median serum IgA level mg/dL, [IQR] (*n* = 103)	34 [12–106]	20 [8–60]	71 [23–134]	0.001
Median serum IgM level mg/dL, [IQR] (*n* = 103)	17 [7–29]	9 [6–18]	24 [15–51]	<0.001
IgM level < 17 mg/dL, *n* (%)	51 (49.5)	36 (69.2)	15 (29.4)	<0.001

Abbreviations: ASCT, autologous stem cell transplantation; BJP, Bence Jones protein; eGFR, estimated glomerular filtration rate; Ig, immunoglobulin; IQR, interquartile range; PD, progressive disease; PR, partial response; SD, stable disease; VGPR, very good PR.

### 3.1 Response to dose 2 of mRNA vaccine

The median duration between dose 2 and TP1 sample collection was 19 days (IQR: 12–33). The median S‐IgG titer of the 109 patients at TP1 was 300 BAU/mL (IQR, 54–1391), and 13 (12%), 42 (38.5%), and 54 (49.5%) patients were categorized as non‐responders, low‐responders, and adequate‐responders, respectively. Multivariable logistic regression analysis revealed that the following factors were significantly associated with becoming adequate‐responders: no active treatment at dose 2 (odds ratio [OR], 11.5; 95% confidence interval [CI], 2.89–45.79), mRNA‐1273 vaccination (OR, 44.18; 95% CI, 5.75–339.77), estimated glomerular filtration rate (eGFR) ≥ 40 mL/min/1.73 m^2^ (OR, 12.86; 95% CI, 2.04–81.98), and IgM level ≥ 17 mg/dL (OR, 14.98; 95% CI, 4.45–50.40) (Table 2).

**TABLE 2:** Univariate and multivariable analyses for factors associated with being adequate‐responders to the second mRNA vaccine dose.

Factor	Cutoff	Univariate analysis		Multivariable analysis	
		Odds ratio (95% CI)	*p*	Odds ratio (95% CI)	*p*
Age	<70 Years	‐			
	≥70 Years	0.46 (0.21–1.00)	0.050		
Sex	Male	‐			
	Female	0.83 (0.39–1.77)	0.630		
Vaccine type	BNT162b2	‐		‐	
	mRNA‐1273	9.60 (1.56–59.01)	0.015	44.18 (5.75–339.77)	<0.001
Anti‐myeloma treatment within 3 months before the second vaccination	Yes	‐		‐	
	No	4.48 (1.72–11.60)	0.002	11.50 (2.89–45.79)	<0.001
Prior ASCT	No	‐			
	Yes	3.25 (1.46–7.23)	0.004		
Lymphocyte count	<1000/μL	‐			
	≥1000/μL	0.69 (0.30–1.58)	0.375		
Serum albumin	<3.5 g/dL	‐			
	≥3.5 g/dL	4.31 (0.95–19.52)	0.058		
eGFR	<40 mL/min/1.73 m^2^	‐		‐	
	≥40 mL/min/1.73 m^2^	2.92 (0.88–9.68)	0.080	12.86 (2.04–81.08)	0.007
Serum IgM level	<17 mg/dL	‐		‐	
	≥17 mg/dL	5.21 (2.25–12.06)	<0.001	14.98 (4.45–50.40)	<0.001

*Note*: Factors with *p* < 0.2 in the univariate analysis were included in the multivariable logistic regression analysis with backward stepwise selection.

Abbreviations: ASCT, autologous stem cell transplantation; CI, confidence interval; eGFR, estimated glomerular filtration rate; Ig, immunoglobulin.

**FIGURE 1:**
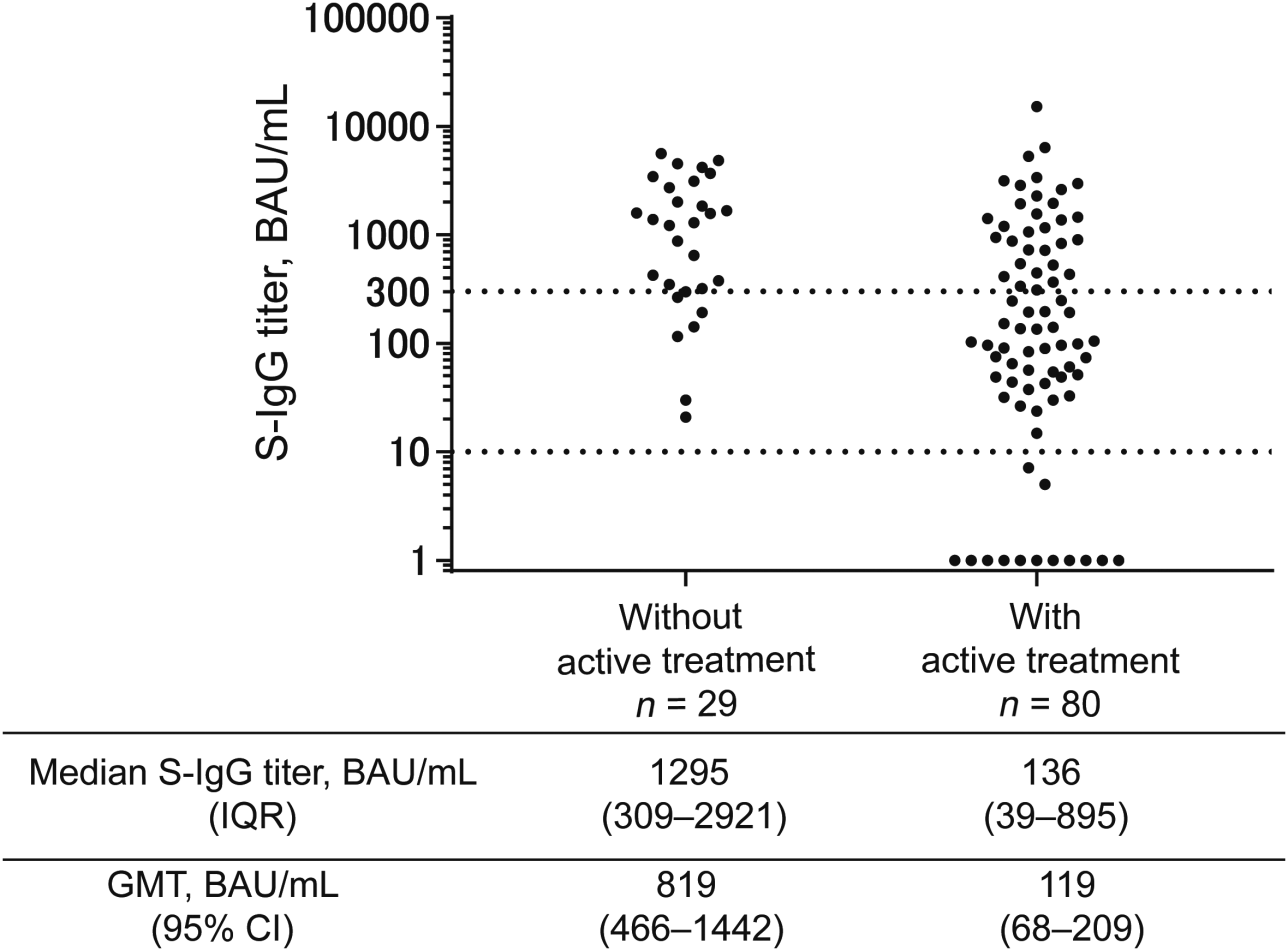
S‐IgG titer (shown in logarithmic scale) after second vaccination in subgroups, based on none or active anti‐myeloma treatment. In this study, active treatment was defined as receiving any anti‐myeloma treatments within 3 months before vaccination. S‐IgG titers of patients without active treatment were significantly higher than those of patients with active treatment (*p* < 0.001). BAU, binding antibody unit; CI, confidence interval; GMT, geometric mean titer; IQR, interquartile range; S‐IgG, immunoglobulin G antibodies against spike proteins.

All patients without active treatment achieved seroconversion, with significantly higher S‐IgG titers than that in patients undergoing active treatment (*p* < 0.001) (Figure 1). Thus, we evaluated the effect of anti‐myeloma treatment subclass on S‐IgG production in patients undergoing active treatment. The anti‐myeloma treatment information of 80 patients is shown in Table S1. Treatment with anti‐CD38 monoclonal antibody (anti‐CD38 mAb) (*p* = 0.261), immunomodulatory drugs (IMiDs) (*p* = 0.816), and proteasome inhibitor (PI) (*p* = 0.636) was not associated with being an adequate‐responder using univariate analysis (Table S1). Furthermore, the geometric mean titer (GMT) of S‐IgG was comparable among the 80 patients treated with or without IMiDs and with or without anti‐CD38 mAb regimens (Figure S3A,B). The GMT was significantly higher in patients treated with PI than in those treated with other regimens (*p* = 0.013) (Figure S3C). However, a higher percentage of patients treated with PI were under two or less lines of therapy compared to those treated with other regimens (*p* = 0.03), which could have resulted in higher S‐IgG levels in patients treated with PI. Furthermore, the S‐IgG titers of individual patients were plotted by the treatment regimen in Figure S3D. Due to other possible confounding factors and small sample size, comparison of S‐IgG titers between treatment regimens was not performed. Notably, all five patients treated with a novel targeted therapy—B‐cell maturation antigen (BCMA) targeted therapy (*n* = 4) or G protein–coupled receptor, class C group 5 member D targeted therapy (*n* = 1)—were non‐responders.

### 3.2 Serial changes in S‐IgG titer over time in patients with seroconversion after dose 2

**FIGURE 2:**
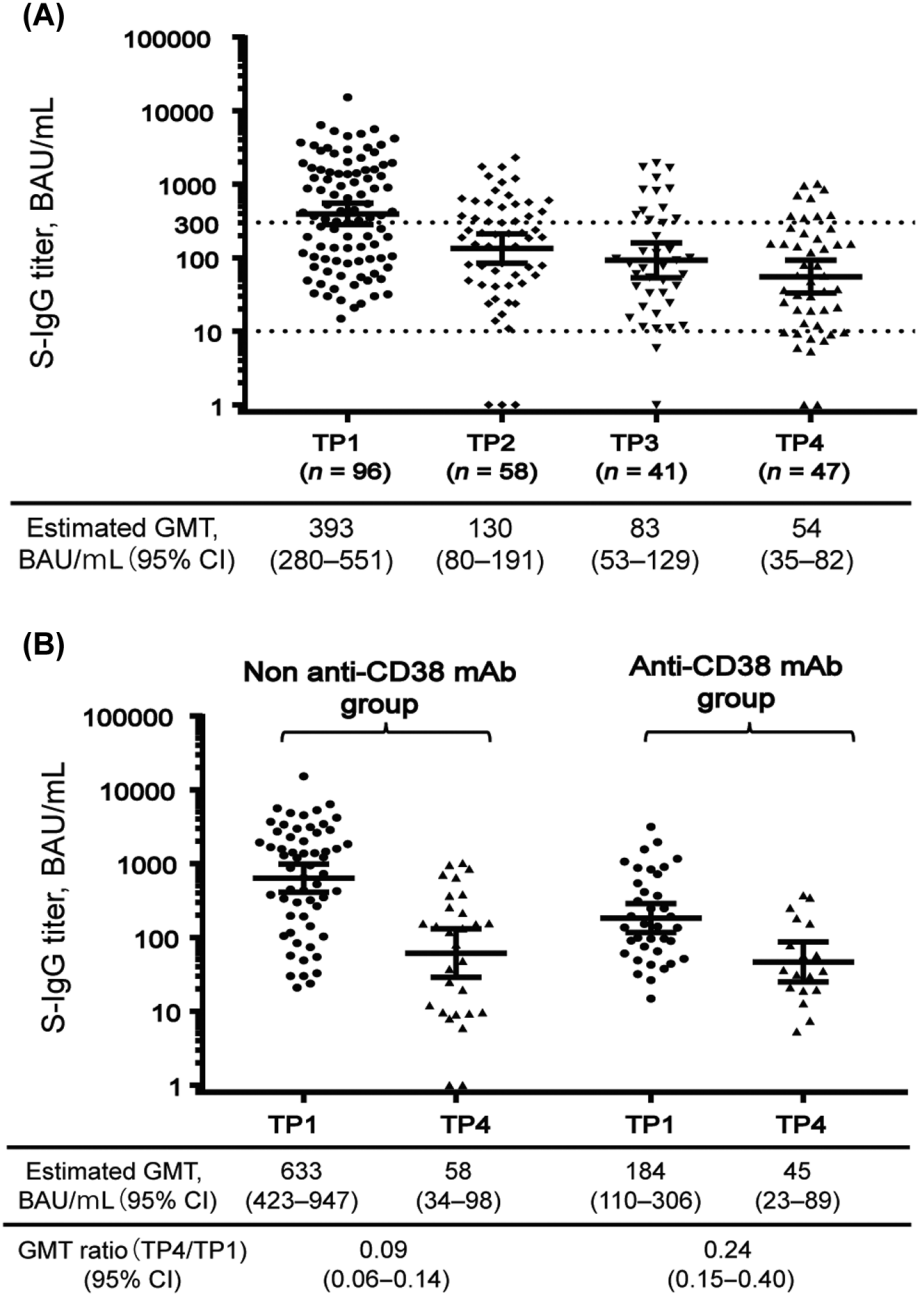
Serial changes in S‐IgG titer (shown in logarithmic scale) over time in patients with seroconversion after the second vaccine dose (*n* = 96). (A) S‐IgG titer was evaluated in TP1 up to TP4. (B) S‐IgG titers of TP1 and TP4 were evaluated in subgroups based on the inclusion or exclusion of active anti‐CD38 monoclonal antibody treatment (defined as administrated within 3 months before the second vaccination). The GMT ratio (TP4/TP1) in S‐IgG titer was significantly lower in patients treated with anti‐CD38 monoclonal antibody (*p* < 0.01). BAU, binding antibody unit; CI, confidence interval; IQR, interquartile range; GMT, geometric mean titer; mAb, monoclonal antibody; S‐IgG, immunoglobulin G antibodies against spike proteins; TP, timepoint; TP1/2/3/4, duration defined as within 7–60 days/91–120 days/121–150 days/151–the day of third vaccine dose, respectively, after the second mRNA vaccine dose.

We evaluated S‐IgG titers in the 96 patients who achieved seroconversion after dose 2 (the GMT was 393 BAU/mL; 95% CI, 280–551), in samples from TP2 up to TP4. We observed decreased S‐IgG titer over time with the estimated GMT 130 BAU/mL at TP2 (GMT ratio to TP1, 0.33), 83 BAU/mL at TP3 (GMT ratio to TP1, 0.21), and 54 BAU/mL at TP4 (GMT ratio to TP1, 0.14), respectively (Figure 2A). In addition, the S‐IgG titer changes in individual patients (58 of the 96 patients) with more than three samples between TP1 and TP4 were described (Figure S4).

Next, we investigated serial changes in S‐IgG titer in the 96 patients, stratified by anti‐CD38 mAb treatment within 3 months prior to dose 2. We observed a mild decrease in GMT over time in patients treated with anti‐CD38 mAb. The GMT ratio of TP4/TP1 was significantly higher in patients treated with anti‐CD38 mAb (0.24 [95% CI, 0.15–0.4]), compared with that in patients treated with regimens other than anti‐CD38 mAb (0.09 [95% CI, 0.06–0.14]) (*p* = 0.004) (Figure 2B). However, the GMT ratio of TP4/TP1 was comparable in patients treated with and without PI regimen (*p* = 0.791), and significantly lower in patients treated with IMiDs regimen than in those treated with other regimens (*p* < 0.001) (Figure S5A,B).

### 3.3 Response to dose 3 of mRNA vaccine (booster vaccination)

Serum samples from 92 patients were available after dose 3, TP5. Of these 92 patients, 20 patients started or changed their treatment following the administration of dose 2 mainly due to disease progression and 7 patients discontinued their treatment following the administration of dose 2. The remaining patients continued on the same regimen after dose 2. The duration between doses 2 and 3 was 220 days (IQR, 207–235). The median gap between dose 3 and sample collection was 20 days (IQR, 12–26), and 69 patients were undergoing active anti‐myeloma treatment.

**FIGURE 3:**
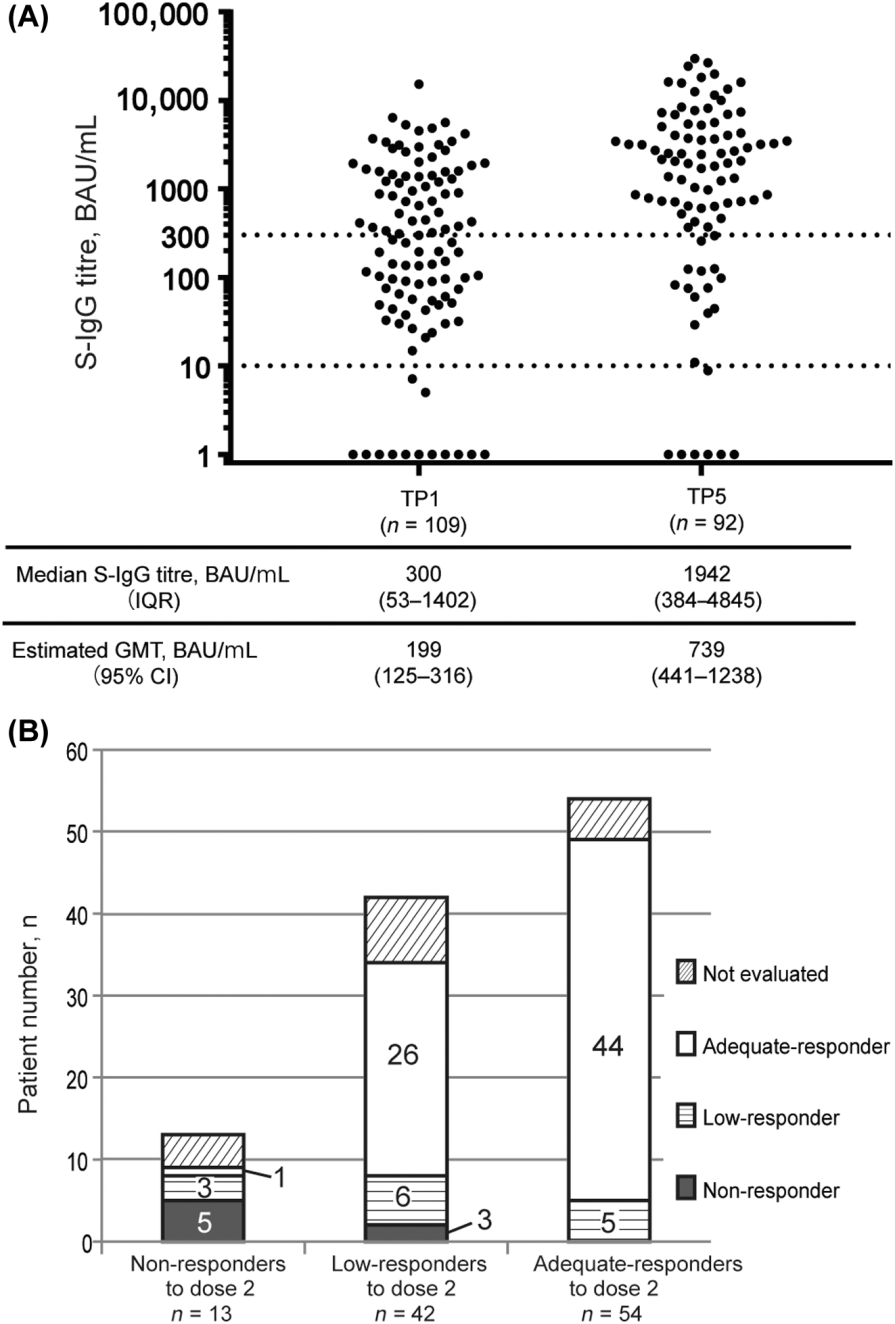
Humoral immune responses after the second and third mRNA vaccination. (A) S‐IgG titer (shown in logarithmic scale) obtained after the second and third mRNA vaccine doses. The GMT was significantly higher at TP5 than that at TP1 (*p* < 0.01). (B) Distribution of responders to the third mRNA vaccine dose according to the response of the second vaccine dose. CI, confidence interval; GMT, geometric mean titer; IQR, interquartile range; S‐IgG, immunoglobulin G antibodies against spike proteins of SARS‐CoV‐2; TP, time point; TP1, duration defined as within 7–60 days after the second mRNA vaccine dose; TP5, duration defined as within 7–60 days after the third mRNA vaccine dose; adequate‐responder, those with an S‐IgG titer ≥300 binding antibody unit (BAU)/mL; low‐responder, one with an S‐IgG titer of 10–300 BAU/mL; non‐responder, one with an S‐IgG titer ≤10 BAU/mL.

The median S‐IgG titer of the 92 patients was 1942 BAU/mL (IQR, 384–4845). The S‐IgG titers obtained after dose 3 were significantly increased compared to those after dose 2 (*p* < 0.001), with a GMT ratio of TP5/TP1, 3.71 (95% CI, 2.53–5.45) (Figure 3A). Based on the S‐IgG titers at TP5, 7 (7.6%), 14 (15.2%), and 71 (77.2%) patients were non‐responders, low‐responders, and adequate‐responders, respectively. Among the nine non‐responders to dose 2, five (56%) achieved seroconversion after dose 3. Among 34 low‐responders to dose 2, 26 (76.5%) became adequate‐responders after dose 3 (Figure 3B). However, among 54 adequate‐responders to dose 2, five (9.3%) became low‐responders to dose 3. The clinical characteristics of these patients are shown in Table S2. Three of these patients received different anti‐myeloma treatments between dose 2 and 3 due to disease progression, and achieved a partial response or better at the time of dose 3. The other two patients showed disease progression after dose 2 but continued on the same regimen after dose 3. In total, 22 patients were low‐ or non‐responders to dose 3. In addition, the S‐IgG titer changes between TP1 and TP5 of individual patients whose samples were collected at both TP1 and TP5 (*n* = 92) are shown in Figure S6. Univariate analysis revealed that vaccine type, serum IgM level, and eGFR were not associated with adequate‐responders to dose 3 (Table S3).

Among 69 patients undergoing active treatment at dose 3, S‐IgG titers based on treatment regimens were plotted and combined with the S‐IgG‐titers of 80 patients undergoing active treatment at dose 2. Patients in all treatment subgroups, except for those in the novel targeted therapy group, demonstrated higher GMT after dose 3. However, no significant difference was observed in GMT after doses 2 and 3 in patients treated with PI ± anti‐CD38 mAb (Figure S7).

### 3.4 Cellular immune response

**FIGURE 4:**
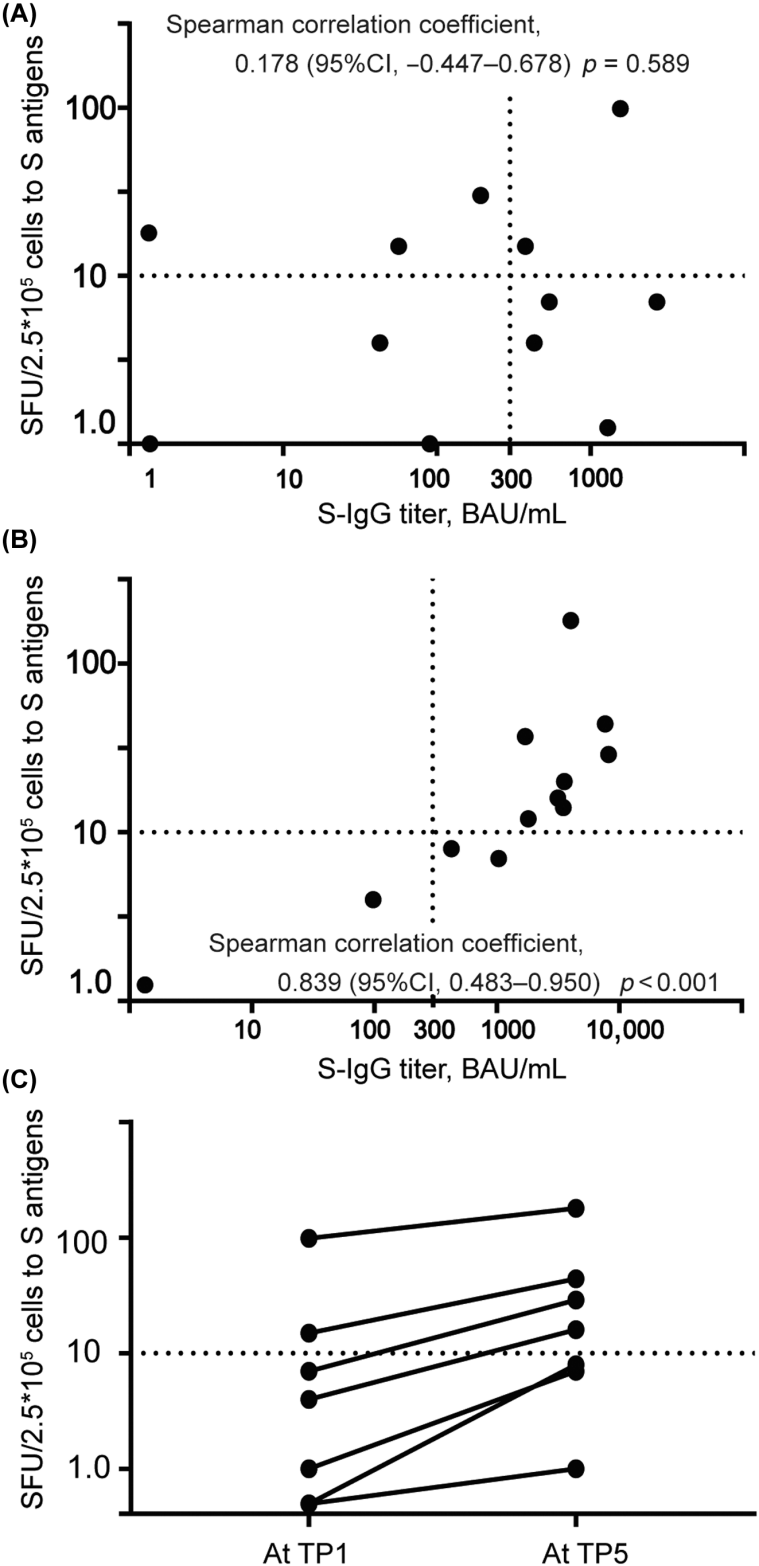
Cellular responses after the second and third mRNA vaccination. (A) The combined results of T‐cell responses to spike antigens (expressed as SFU, shown in logarithmic scale) and S‐IgG titer (shown in logarithmic scale) after the second mRNA vaccination (*n* = 12). There was no correlation between SFU and S‐IgG titer (*p* = 0.589). (B) The combined results of T‐cell responses to spike antigens and S‐IgG titer after the third mRNA vaccination (*n* = 12). There was a significant correlation between SFU and S‐IgG titer (*p* < 0.001). (C) T‐cell response to spike antigens in the paired samples obtained at TP1 and TP5 (*n* = 7). The number of SFU was significantly larger at TP5 (*p* < 0.01). CI, confidence interval; SFU, spot‐forming unit; S‐IgG, immunoglobulin G antibodies against spike proteins of SARS‐CoV‐2; TP, timepoint; TP1, duration defined as within 7–60 days after the second mRNA vaccine dose; TP5, duration defined as within 7–60 days after the third mRNA vaccine dose.

For the T‐SPOT assay, 12 PBMC samples at TP1 and TP5 from 17 patients and 7 paired samples were available. Table S4 shows detailed information of these patients. We observed that five (42%) and seven (64%) patients were cellular responders after doses 2 and 3, respectively. The representative results of the T‐SPOT assay are shown in Figure S8. At dose 2, three cellular responders were non‐ or low‐responders, and four cellular non‐responders were adequate‐responders, suggesting that humoral immunity might function independently of cellular immunity and vice versa (Figure 4A). By contrast, the correlation between cellular and humoral immune responses increased after dose 3 (Figure 4B). We observed a significant correlation between cellular and humoral immune responses at TP5 (Spearman correlation coefficient, 0.839 [*p* < 0.001]), and not at TP1 (Spearman correlation coefficient, 0.178 [*p* = 0.589]). Moreover, the SFU against the spike antigens was significantly increased at TP5 than that at TP1 in the seven paired samples (*p* < 0.01) (Figure 4C).

## 4 DISCUSSION

We evaluated the humoral and cellular response induced by the novel SARS‐CoV‐2 mRNA vaccines in patients with PCD. We observed that most patients obtained higher S‐IgG titers after dose 3 compared to dose 2, including patients receiving any drug subclasses other than novel‐targeted therapy. Additionally, the T‐SPOT assay demonstrated increased cellular response after dose 3, thus highlighting the significance of booster mRNA vaccination in patients with PCD, the representative immunocompromised population.

We found that the vaccine type (BNT162b2), active treatment (anti‐myeloma treatments within 3 months prior to vaccination), eGFR < 40 mL/min/1.73 m^2^, and serum IgM < 17 mg/dL were associated with non‐adequate‐responders, consistent with results of previous studies.^19^, ^22^, ^23^, ^24^ Importantly, the negative effects of these factors were attenuated after dose 3 (booster vaccination), suggesting that the booster vaccination could partly overcome the negative factors in acquiring an adequate humoral response.

In addition, drug subclass, including PI, IMiDs, and anti‐CD38 mAb, were not negatively associated with being adequate‐responders or S‐IgG production level, in restricted patients undergoing anti‐myeloma treatment. Previous studies reported that anti‐CD38 mAb might negatively affect S‐IgG production.^9^, ^25^, ^26^ However, several confounding factors could affect S‐IgG production after anti‐CD38 mAb treatment. The S‐IgG titers of patients treated with anti‐CD38 mAb would be lower than those without anti‐CD38 mAb if including patients not undergoing active treatment. Moreover, anti‐CD38 mAb treatment is usually used concurrently with IMiDs or PI, which would further affect S‐IgG production. We observed that patients treated with anti‐CD38 mAb maintenance monotherapy, showed relatively higher titers (Figure S3D), supporting that anti‐CD38 mAb itself might not be a negative factor in humoral immune response.

Terao et al. reported that depletion of CD38+ regulatory T (T reg) cells by anti‐CD38 mAb induced a durable response to mRNA vaccination, with high antibody titers 4–12 weeks after vaccination in patients with lower CD38+ T reg levels but decreased titers in patients with higher CD38+ T reg cell levels.^27^ Similarly, we observed a smaller decrease in S‐IgG titer (TP4/TP1) in patients under active treatment with anti‐CD38 mAb compared to that in patients without anti‐CD38 mAb therapy. These observations support a need for further research on the impact of anti‐CD38 mAb in inducing and maintaining humoral response to mRNA vaccines.

In this study, the T‐SPOT assay was used to examine vaccine‐induced cellular immune response. T‐SPOT is a simplified enzyme‐linked immunosorbent spot (ELISPOT) assay, which identifies T cells in peripheral blood that release IFN‐γ in response to SARS‐CoV‐2 antigen stimulation. The T‐SPOT assay is performed using commercial kits with standardized methodology, making the results more generalizable. We observed a correlation between the humoral and cellular responses in TP5 samples, which was not observed in TP1 samples. Enßle et al. reported that a clear correlation between humoral and cellular (examined via ELISPOT) immune responses was not observed in patients with myeloma after dose 2; however, a positive correlation was observed in healthy individuals.^28^ A similar trend was observed in another study, which evaluated IFN‐γ and Interleukin‐2 responses to SARS‐CoV‐2 antigen stimulation in PBMC from healthy individuals and patients with various types of hematological malignancies who received two doses of BNT162b2.^12^ The study reported that cellular and humoral responses were correlated in healthy individuals but not in patients with a hematological malignancy, although statistical analysis was not performed.^12^ Consistent with these studies, we observed that the third (booster) vaccination in patients with PCD enhanced both humoral and cellular immune responses, resulting in similar immune‐responses as those observed in healthy individuals. Because the T‐SPOT assay is not available in clinical practice, surrogate markers that are more widely available are preferable to predict the achievement of cellular immune response to mRNA vaccination. Peripheral blood CD4 T‐cell count may be such a candidate,^29^ although further investigation is warranted.

Patients who underwent BCMA‐targeted therapy did not obtain adequate humoral response even after dose 3. A previous study reported that 12 of 165 (7.3%) patients included in a phase 1–2 trial that evaluated the efficacy and safety of teclistamab, a BCMA‐targeted bispecific antibody, died due to COVID‐19‐associated adverse events.^30^ This implies that BCMA‐targeted therapy might have negative effects on immunity acquisition via SARS‐CoV‐2 vaccination. The impact of BCMA‐targeted therapy on vaccine‐induced cellular immunity should be further investigated; this could not be elucidated in our study due to a small sample size.

Our study had several limitations. First, the timing of the sample collection was not uniform. However, increasing patient visits to the hospital for sample harvesting for this study was ethically unacceptable during the ongoing COVID‐19 pandemic. Second, the T‐SPOT assay could only be performed with samples containing a sufficient number of cells, possibly resulting in a selection bias for positive factors in being cellular responders. Furthermore, cryopreserved cells were used for the T‐SPOT assay, which was not originally supported by the T‐SPOT kit. However, several studies reported the applicability of cryopreserved cells for ELISPOT or T‐SPOT assay,^28^, ^31^, ^32^ and using cryopreserved cells allowed us to perform the tests at the same time under uniform conditions.

## 5 CONCLUSION

This study highlighted the significance of booster SARS‐CoV‐2 mRNA vaccination in patients with PCD with respect to humoral and cellular immunity. Moreover, this study suggested the potential impact of drug subclasses on vaccine‐induced humoral immune response.

## AUTHOR CONTRIBUTIONS

**Tomotaka Suzuki:** Conceptualization (lead); data curation (lead); formal analysis (lead); investigation (equal); methodology (equal); project administration (equal); writing – original draft (lead). **Shigeru Kusumoto:** Funding acquisition (equal); investigation (equal); methodology (equal); project administration (equal); supervision (lead); writing – review and editing (lead). **Yoshiko Kamezaki:** Formal analysis (equal); resources (equal). **Hiroya Hashimoto:** Formal analysis (equal); validation (lead); writing – review and editing (equal). **Nozomi Nishitarumizu:** Investigation (equal); writing – review and editing (equal). **Yoko Nakanishi:** Investigation (equal); writing – review and editing (equal). **Yukiyasu Kato:** Investigation (equal); writing – review and editing (equal). **Akimi Kawai:** Investigation (equal); writing – review and editing (equal). **Naohiro Matsunaga:** Investigation (equal); writing – review and editing (equal). **Toru Ebina:** Investigation (equal); writing – review and editing (equal). **Tomoyuki Nakamura:** Investigation (equal); writing – review and editing (equal). **Yoshiaki Marumo:** Investigation (equal); writing – review and editing (equal). **Kana Oiwa:** Investigation (equal); writing – review and editing (equal). **Shiori Kinoshita:** Investigation (equal); writing – review and editing (equal). **Tomoko Narita:** Investigation (equal); writing – review and editing (equal). **Asahi Ito:** Investigation (equal); writing – review and editing (equal). **Atsushi Inagaki:** Investigation (equal); writing – review and editing (equal). **Masaki Ri:** Investigation (equal); writing – review and editing (equal). **Hirokazu Komatsu:** Investigation (equal); writing – review and editing (equal). **Takashi Aritsu:** Funding acquisition (equal); resources (lead); writing – review and editing (equal). **Shinsuke Iida:** Funding acquisition (equal); investigation (equal); resources (equal); writing – review and editing (equal).

## CONFLICT OF INTEREST STATEMENT

S. Kusumoto reports research funding from Daiichi Sankyo and Chugai Pharmaceutical and honoraria from Chugai Pharmaceutical. M. Ri reports research funding from Bristol‐Myers Squibb/Celgene, Daiichi Sankyo, Chugai Pharmaceutical, Kyowa Kirin, Ono Pharmaceutical and Takeda and honoraria from Janssen. S. Iida reports research funding, consulting fees, and honoraria from Janssen, Takeda, and Sanofi; research funding and honoraria from Bristol‐Myers Squibb/Celgene and Ono Pharmaceutical; research funding and consulting fees from Pfizer; and research funding from AbbVie, Amgen, Daiichi Sankyo, Otsuka, and Caelum. In addition, S. Iida is an associate editor of Cancer Science.

## ETHICS STATEMENT

*Approval of the research protocol by an Institutional Reviewer Board*: This study was approved by the Institutional Review Board of the NCUH and was conducted in accordance with the principles of the Declaration of Helsinki. *Informed consent*: Written informed consent was obtained from all participants to store and use their blood samples. *Registry and the Registration no. of the study/trial*: N/A. *Animal studies*: N/A.

## CLINICAL TRIAL REGISTRATION

N/A.

## Supporting information


Data S1.

## Data Availability

The datasets generated and/or analyzed during the current study are available from the corresponding author upon reasonable request.
