# Supplementary material for: Humoral and cellular immune response to second and third severe acute respiratory syndrome coronavirus 2 mRNA vaccine in patients with plasma cell dyscrasia
Source: Cancer Med. 2023 Apr 26;12(12):13135–44. doi: 10.1002/cam4.5996 (PMC10315730; doi:10.1002/cam4.5996)
Supplement: Supplementary file 1 — Data S1. [file CAM4-12-13135-s001.zip › CAM4_5996_Fig_S1 afterR.docx]

**
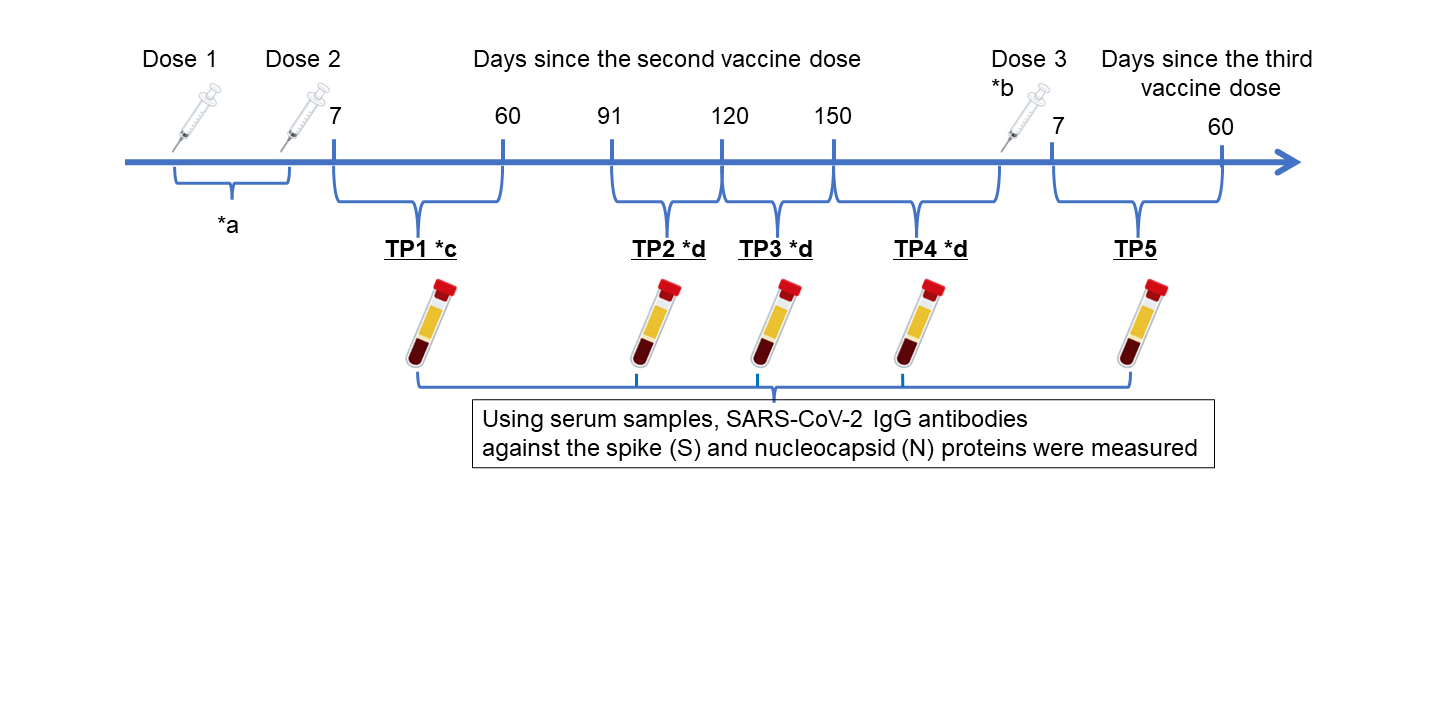
**

**Fig S1.** The schema of the serum sample collection.

The timing of serum sample collection depended on the timing of the patient’s visit to the Nagoya City University Hospital for a regular check-up of plasma cell dyscrasia.

*a：The duration between dose 1 and 2 was 21 days and 28 days for BNT162b2 and mRNA-1273, respectively. All patients received mRNA vaccination according to the manufacturer’s instructions at a local clinic or group vaccination venue.

*b：The third mRNA vaccination was allowed 6 months after the second dose in Japan.

*c：Serum samples obtained at time point (TP) 1 according to the eligibility criteria.

*d：Serum samples obtained at TP2, TP3, and TP4 were evaluated only in patients who demonstrated seroconversion at TP1.
