# Supplementary material for: Humoral and cellular immune response to second and third severe acute respiratory syndrome coronavirus 2 mRNA vaccine in patients with plasma cell dyscrasia
Source: Cancer Med. 2023 Apr 26;12(12):13135–44. doi: 10.1002/cam4.5996 (PMC10315730; doi:10.1002/cam4.5996)
Supplement: Supplementary file 1 — Data S1. [file CAM4-12-13135-s001.zip › CAM4_5996_Fig_S2 for revise.docx]

**
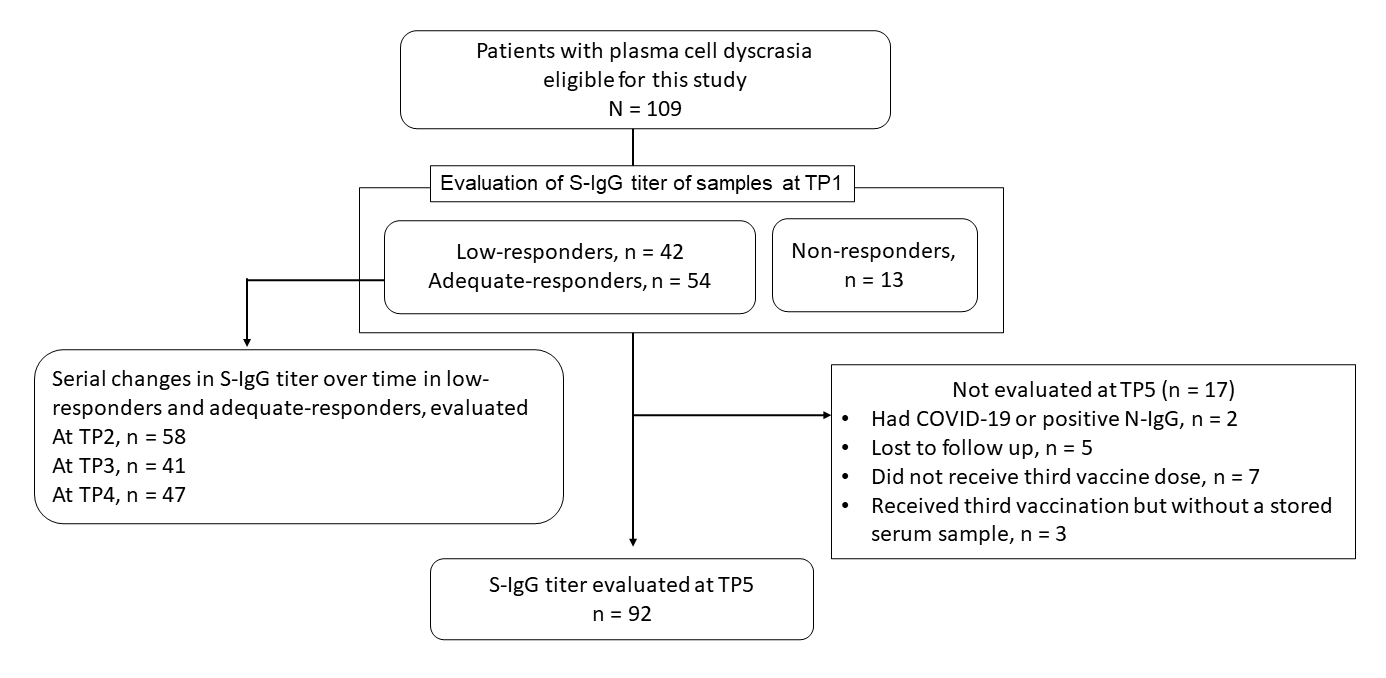
**

**Fig S2.** Patient consort diagram

Patients with a known history of SARS-CoV-2 infection before dose 2 were excluded from this study. No patient was positive for N-IgG in samples at TP1. The results of SARS-CoV-2 IgG antibodies against spike proteins (S-IgG) titer in patients who showed positive SARS-CoV-2 IgG antibodies against nucleocapsid proteins (N-IgG) titers in samples at TP1 or later, or in those who were clinically diagnosed with COVID-19 after dose 2 were not used for further analyses.

TP, time point; IQR, interquartile range; TP1, duration defined as within 7 to 60 days after the second mRNA vaccine dose (median duration between second vaccination and sample collection, 19 days [IQR, 12–33]); TP2, duration defined as within 91 to 120 days after the second mRNA vaccine dose (median duration between second vaccination and sample collection, 101 days [IQR, 96–109]); TP3, duration defined as within 121–150 days after the second mRNA vaccine dose (median duration between second vaccination and sample collection, 134 days [IQR, 129–145]); TP4, duration defined as within 151 days after the second mRNA vaccine dose until the third vaccine dose (median duration between second vaccination and sample collection, 170 days [IQR, 162–190]); TP5, duration defined as within 7 to 60 days after the third mRNA vaccine dose (median duration between third vaccination and sample collection, 20 days [IQR, 12–26]); adequate-responder, one with an S-IgG titer ≥ 300 binding antibody unit (BAU)/mL; low-responder, one with an S-IgG titer of 11–300 BAU/mL; non-responder, one with an S-IgG titer ≤ 10 BAU/mL.
