# Supplementary material for: Humoral and cellular immune response to second and third severe acute respiratory syndrome coronavirus 2 mRNA vaccine in patients with plasma cell dyscrasia
Source: Cancer Med. 2023 Apr 26;12(12):13135–44. doi: 10.1002/cam4.5996 (PMC10315730; doi:10.1002/cam4.5996)
Supplement: Supplementary file 1 — Data S1. [file CAM4-12-13135-s001.zip › CAM4_5996_Fig_S3_r_clean copy.docx]

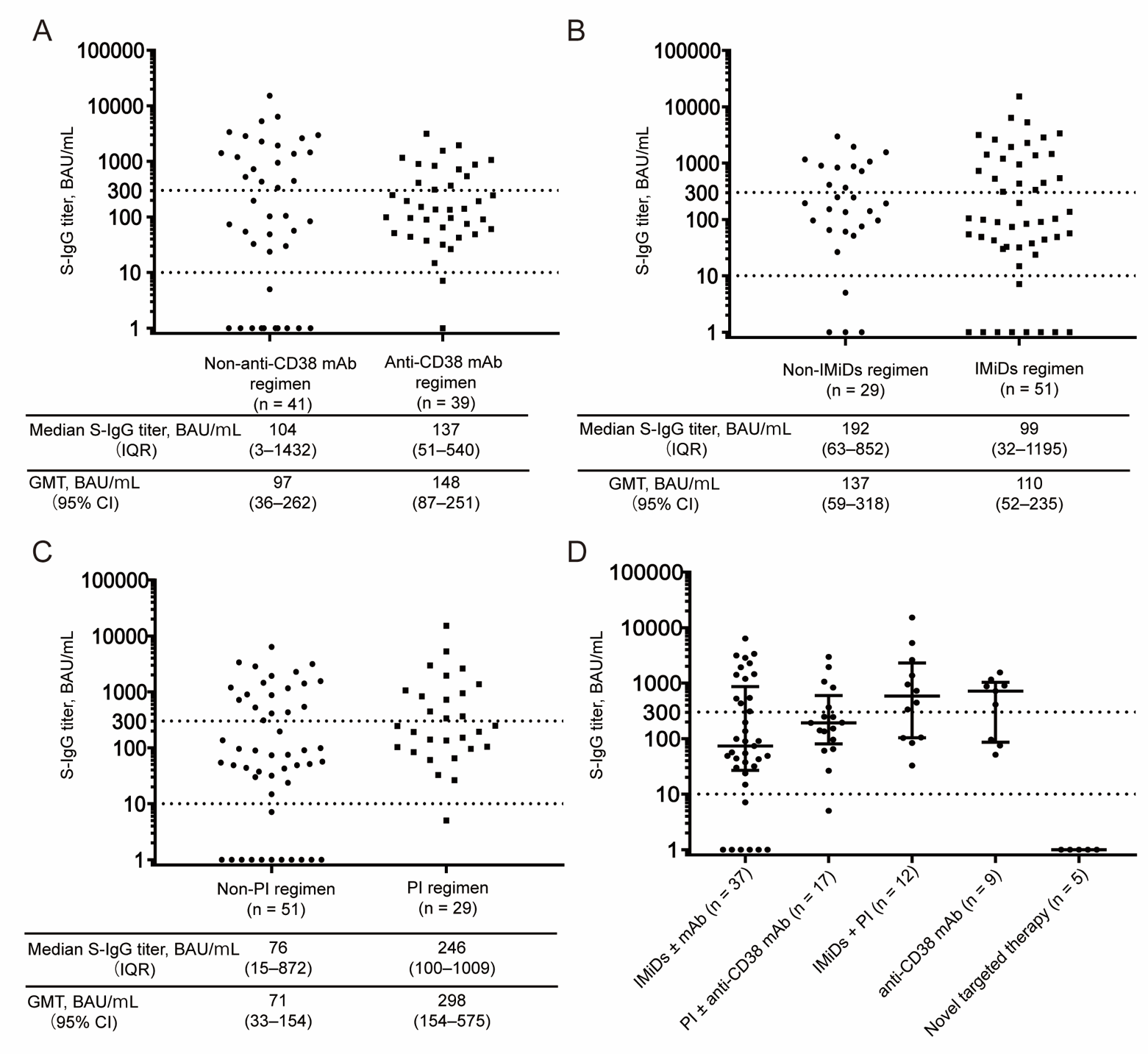


**Fig S3**. S-IgG titer (shown in logarithmic scale) after the second mRNA vaccination in patients undergoing active anti-myeloma treatment* (n = 80).

(A) S-IgG titer of patients treated with or without anti-CD38 monoclonal antibody. The GMTs between the two groups were not significantly different (p = 0.71). (B) S-IgG titer of patients treated with or without immunomodulatory drug. The GMTs between the two groups were not significantly different (p = 0.46). (C) S-IgG titer of patients treated with or without proteasome inhibitor. The GMT was significantly higher in patients treated with proteasome inhibitor (p = 0.013) (D) S-IgG titer of patients according to treatment regimens.

S-IgG, immunoglobulin G antibodies against spike proteins; CI, confidence interval; BAU, binding antibody unit; IQR, interquartile range; GMT, geometric mean titer; mAb, monoclonal antibody; IMiDs, immunomodulatory drugs; PI, proteasome inhibitor; Novel targeted therapy, B-cell maturation antigen targeted bispecific antibody or antibody-drug conjugate (n = 4) and G protein–coupled receptor, class C group 5 member D targeted bispecific antibody (n = 1). *Active anti-myeloma treatment is defined as any anti-myeloma treatment within 3 months before vaccination.
