# Supplementary material for: Humoral and cellular immune response to second and third severe acute respiratory syndrome coronavirus 2 mRNA vaccine in patients with plasma cell dyscrasia
Source: Cancer Med. 2023 Apr 26;12(12):13135–44. doi: 10.1002/cam4.5996 (PMC10315730; doi:10.1002/cam4.5996)
Supplement: Supplementary file 1 — Data S1. [file CAM4-12-13135-s001.zip › CAM4_5996_Fig_S4_r_clean copy.docx]

**Fig S4.** Changes in S-IgG titer (shown in logarithmic scale) over time in individual patients who achieved seroconversion (n=58)

This line graph shows S-IgG titer changes from patients (n=58) who achieved seroconversion after the second vaccination and had more than three samples available between TP1 and TP4.

S-IgG, immunoglobulin G antibodies against spike proteins; BAU, binding antibody unit; TP, time point, TP1, duration defined as within 7 to 60 days after the second mRNA vaccine dose; TP2, duration defined as within 91 to 120 days after the second mRNA vaccine dose; TP3, duration defined as within 121 to 150 days after the second mRNA vaccine dose; TP4, duration defined as within 151 days after the second mRNA vaccine dose until the third vaccine dose.
