# Supplementary material for: Humoral and cellular immune response to second and third severe acute respiratory syndrome coronavirus 2 mRNA vaccine in patients with plasma cell dyscrasia
Source: Cancer Med. 2023 Apr 26;12(12):13135–44. doi: 10.1002/cam4.5996 (PMC10315730; doi:10.1002/cam4.5996)
Supplement: Supplementary file 1 — Data S1. [file CAM4-12-13135-s001.zip › CAM4_5996_Fig_S5_r_clean copy.docx]

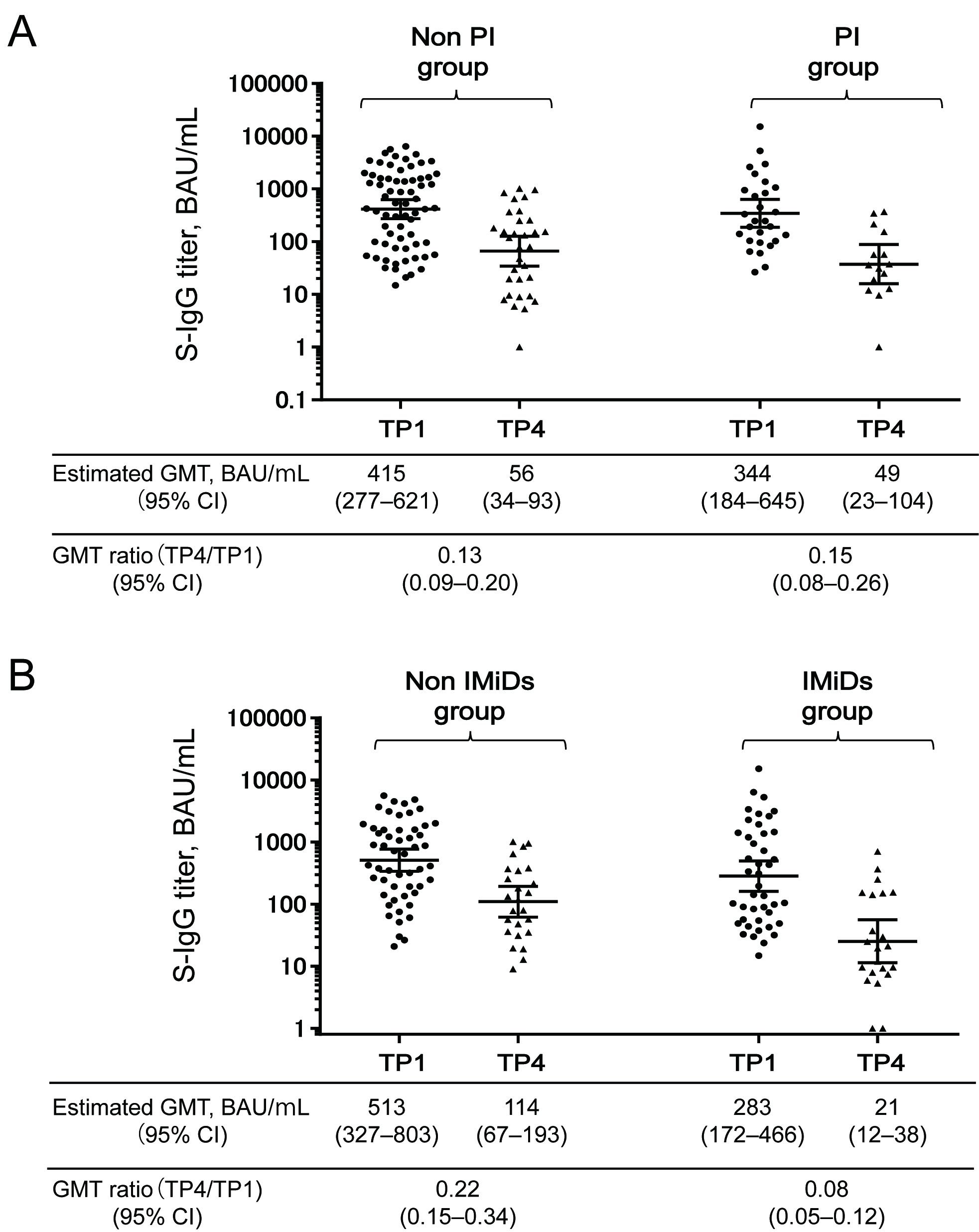


**Fig S5.** Changes in S-IgG titer (shown in logarithmic scale) over time in subgroups of patients according to anti-myeloma treatments.

(A) S-IgG titer of patients treated with or without proteasome inhibitor. The GMT ratio (TP4/TP1) was comparable between the groups (p=0.79) (B) S-IgG titer of patients treated with or without immunomodulatory drug. The GMT ratio (TP4/TP1) was significantly lower in patients treated with IMiDs (p < 0.001).

S-IgG, immunoglobulin G antibodies against spike proteins; BAU, binding antibody unit; GMT, geometric mean titer; CI, confidence interval; PI, proteasome inhibitor; IMiDs, immunomodulatory drugs; TP, time point, TP1, duration defined as within 7 to 60 days after the second mRNA vaccine dose; TP4, duration defined as within 151 days after the second mRNA vaccine dose until the third vaccine dose.
