# Supplementary material for: Humoral and cellular immune response to second and third severe acute respiratory syndrome coronavirus 2 mRNA vaccine in patients with plasma cell dyscrasia
Source: Cancer Med. 2023 Apr 26;12(12):13135–44. doi: 10.1002/cam4.5996 (PMC10315730; doi:10.1002/cam4.5996)
Supplement: Supplementary file 1 — Data S1. [file CAM4-12-13135-s001.zip › CAM4_5996_Fig_S6r_clean copy.docx]

**Fig S6.** Changes in S-IgG titer (shown in logarithmic scale) in individual patients who had both TP1 and TP5 samples collected (n=92)

S-IgG, immunoglobulin G antibodies against spike proteins; BAU, binding antibody unit; TP, time point, TP1, duration defined as within 7 to 60 days after the second mRNA vaccine dose; TP5, duration defined as within 7 to 60 days after the third mRNA vaccine dose
