# Supplementary material for: Humoral and cellular immune response to second and third severe acute respiratory syndrome coronavirus 2 mRNA vaccine in patients with plasma cell dyscrasia
Source: Cancer Med. 2023 Apr 26;12(12):13135–44. doi: 10.1002/cam4.5996 (PMC10315730; doi:10.1002/cam4.5996)
Supplement: Supplementary file 1 — Data S1. [file CAM4-12-13135-s001.zip › CAM4_5996_Fig_S7_r_clean copy.docx]

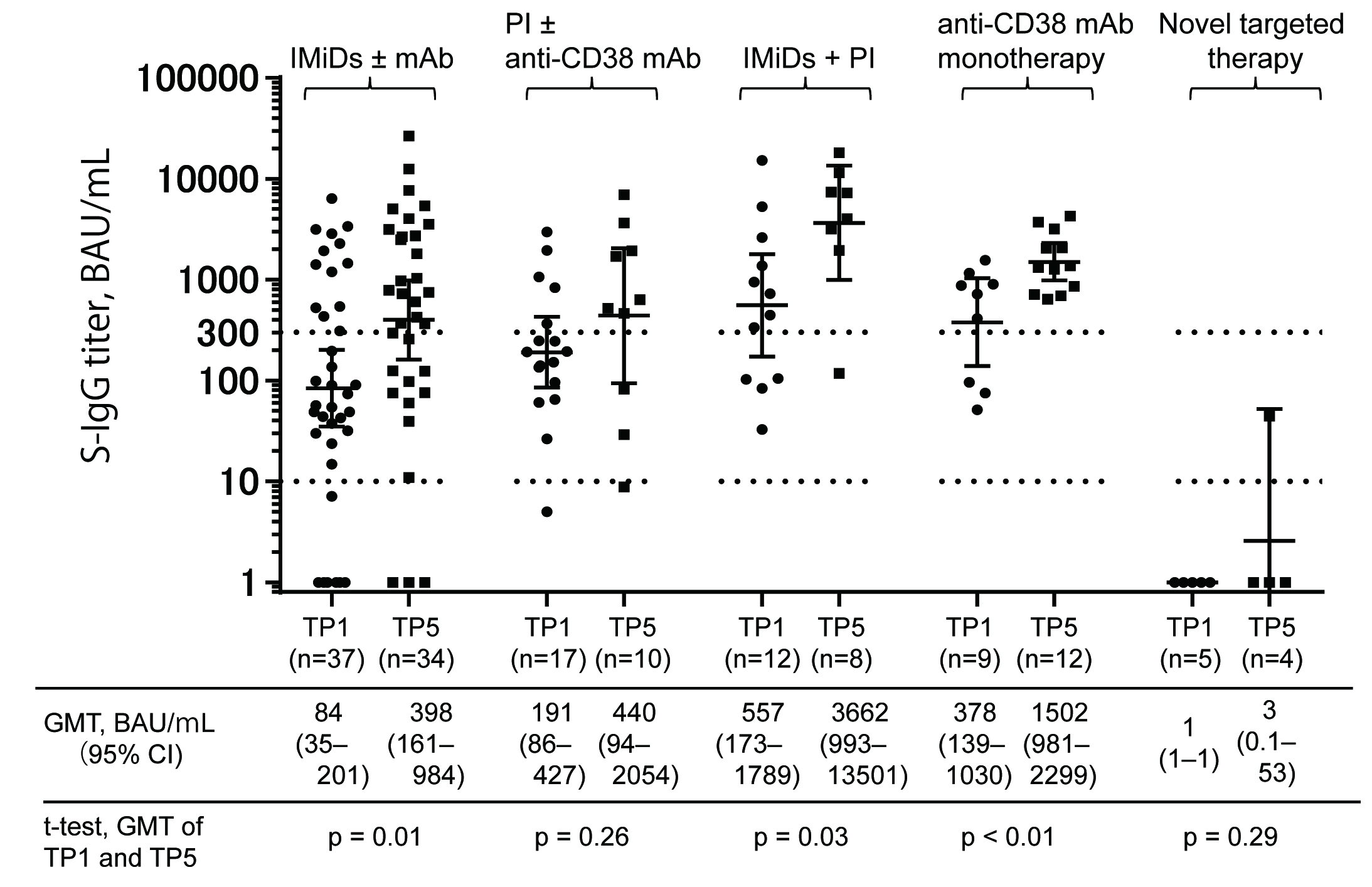
 **Fig S7.** S-IgG titers (shown in logarithmic scale) after the second and third mRNA vaccination in subgroups of patients according to anti-myeloma treatments.

TP, time point; TP1, duration defined as within 7 to 60 days after the second mRNA vaccine dose; TP5, duration defined as within 7 to 60 days after the third mRNA vaccine dose; S-IgG, immunoglobulin G antibodies against spike proteins; CI, confidence interval; BAU, binding antibody unit; IQR, interquartile range; GMT, geometric mean titer; mAb, monoclonal antibody; IMiDs, immunomodulatory drugs; PI, proteasome inhibitor; Novel targeted therapy, targeted therapy against B-cell maturation antigen (n = 4 at TP1 and TP5) or G protein–coupled receptor, class C group 5 member D (n =1 and n = 0 at TP1 and TP5, respectively).
