# Supplementary material for: Humoral and cellular immune response to second and third severe acute respiratory syndrome coronavirus 2 mRNA vaccine in patients with plasma cell dyscrasia
Source: Cancer Med. 2023 Apr 26;12(12):13135–44. doi: 10.1002/cam4.5996 (PMC10315730; doi:10.1002/cam4.5996)
Supplement: Supplementary file 1 — Data S1. [file CAM4-12-13135-s001.zip › CAM4_5996_Fig_S8 for revise.docx]

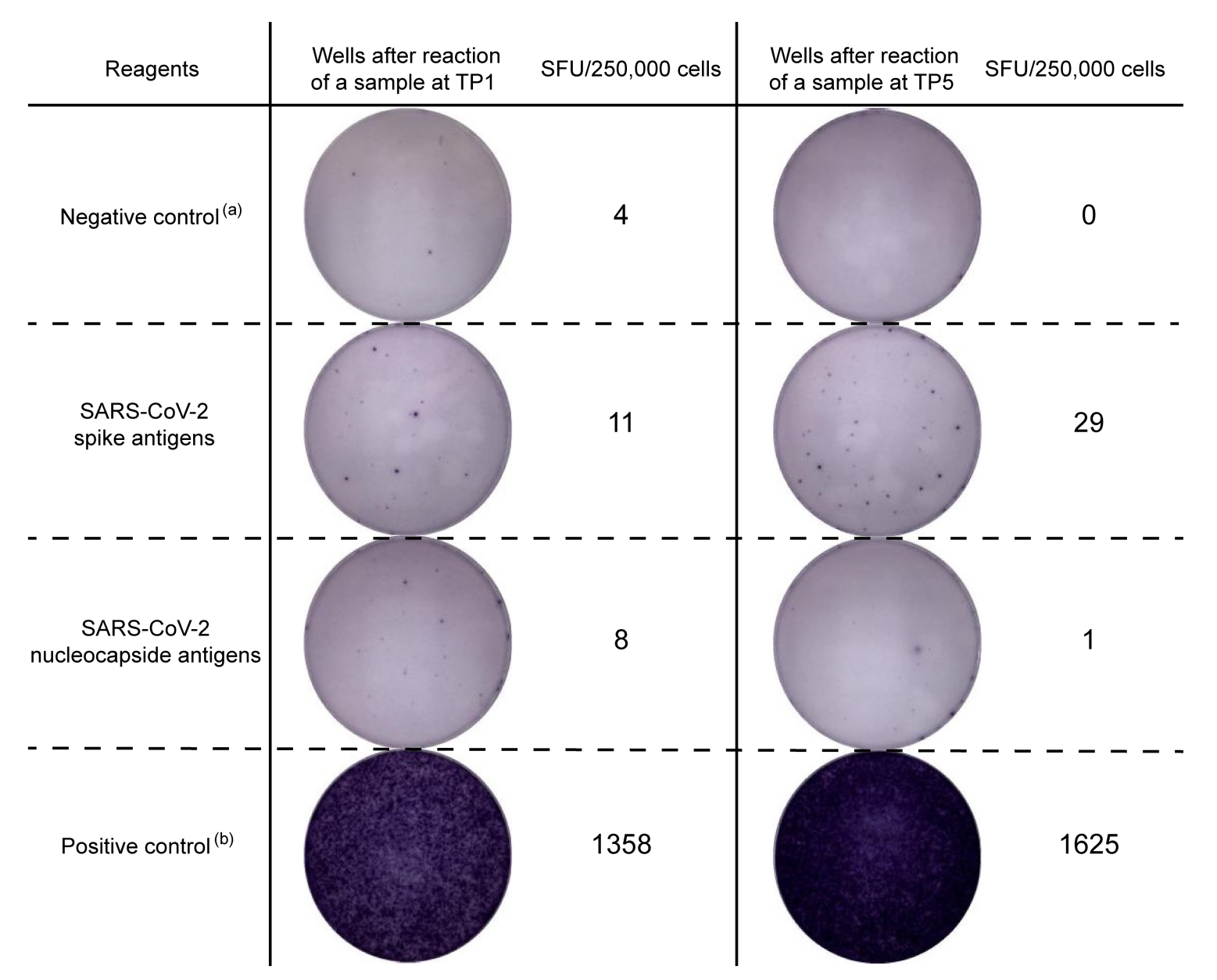


**Fig S8.** Representative results of the T-SPOT assay obtained from a patient at TP1 and TP5

For evaluating T-cell response against SARS-CoV-2 antigens, the spot forming unit (SFU) per 250,000 cells from the negative control wells was subtracted from the SFU in the antigen wells. Therefore, the SFU in spike and nucleocapside antigen wells in the sample at TP1 was calculated as seven and four, respectively. The cellular response in the sample at TP1 was determined as negative, whereas the cellular response in the sample was positive at TP5 (SFU, 29) (the cut-off value of determining positive cellular response, ≥ 10 SFU per 250,000 cells in spike protein well).

(a) Negative control includes Roswell Park Memorial Institute-1640 media. (b) Positive control includes phytohemagglutin.

TP, time point; TP1, duration defined as within 7 to 60 days after the second mRNA vaccine dose; TP5, duration defined as within 7 to 60 days after the third mRNA vaccine dose; SFU, spot forming unit.
