# Supplementary material for: Humoral and cellular immune response to second and third severe acute respiratory syndrome coronavirus 2 mRNA vaccine in patients with plasma cell dyscrasia
Source: Cancer Med. 2023 Apr 26;12(12):13135–44. doi: 10.1002/cam4.5996 (PMC10315730; doi:10.1002/cam4.5996)
Supplement: Supplementary file 1 — Data S1. [file CAM4-12-13135-s001.zip › CAM4_5996_Supplemantal_methods_clean copy.docx]

**Supplemental methods**

**Cut-off value of SARS-CoV-2 IgG antibodies against nucleocapsid proteins**

Yazaki et al. (JAMA Oncol. 2021; 7:1141-48) evaluated the serum SARS-CoV-2 antibody status acquired by SARS-CoV-2 infection, but not by vaccination, in patients with cancer and healthcare workers in Japan. They used the same measurement system as ours to evaluate SARS-CoV-2 IgG antibodies against spike (S) and nucleocapsid proteins (N-IgG).

They used 60 samples of convalescent plasma from patients with COVID-19, confirmed by reverse transcription polymerase chain reaction and 500 negative control samples. The area under the curve was determined by analyzing the receiver operating characteristic curve. Thus, the cut-off value of the positive N-IgG titer was determined to be 15.3 SU/mL, demonstrating 100% sensitivity and 99.8% specificity.

However, because our study participants were patients with malignant lymphoma, with decreased antibody production ability, the cut-off value of positive N-IgG was set at 10.0 SU/mL, lower than that set by Yazaki et al.

In this study, patients with an N-IgG titer ≥ 10 SU/mL at TP1 were regarded as having prior SARS-CoV-2 infection and were excluded from this study; however, no patient in our cohort demonstrated positive N-IgG titer at TP1.

**Evaluation of Cellular immune response**

1. Preparation of cryopreserved peripheral blood mononuclear cell (PBMC)

Fresh whole blood samples from patients were cryopreserved using the methods described below. Fresh whole blood (5 mL) was collected in heparin tubes layered over Ficoll-Paque PLUS® (Cytiva, Marlborough, MA, USA) in Falcon tubes and centrifuged at 1500 rpm for 30 min at room temperature. The PBMC fraction was collected and washed twice in cold phosphate-buffered saline (PBS). Total cell counts were calculated using the NucleoCounter® system (ChemoMetec, Denmark). The PBMC fraction was diluted with 1 mL of Cellbanker1 (Nippon Zenyaku Kogyo CO., LTD) and cryopreserved at -80 °C. Isolation and cryopreservation of PBMC were performed by laboratory technicians at the Nagoya City University Hospital.

1. Evaluation of vaccine-induced cellular immune response

The T-SPOT® Discovery SARS-CoV-2 kit (T-SPOT assay) (Oxford Immunotec, Oxford, United Kingdom) was performed to evaluate vaccine-induced cellular immune response. The assay was performed at Sysmex Corporation (Kobe, Hyogo, Japan), in a blinded manner.

Frozen vials were thawed quickly using a 37 °C water bath for 10 min. Thawed PBMCs were subsequently washed three time in PBS by centrifugation (600 ×*g* for 7 min, twice and 350 ×*g* for 3 min, once), and resuspended in AIM-V serum free tissue culture media. Total cell counts were calculated using a Countess™ 3 Automated Cell Counter (Thermo Fisher Scientific, Waltham, MA, USA) and PBMCs were placed at 37 ºC in a 5% CO_2_ incubator for more than 2 h for resting.

We followed the assay protocol as outlined in the product instructions for freshly isolated PBMCs. The kit, composed of four panels, consisted of the SARS-CoV-2 spike antigens, nucleocapsid antigens, membrane antigens, and the high-homology regions of the coronaviruses, respectively. However, we only used the spike- and nucleocapsid- antigen panels along with negative and positive controls due to limited number of cells available, and these two panels were considered sufficient to evaluate the vaccine-induced immune response to SARS-CoV-2.

A total of 250,000 cells per well were conditioned and plated into individual wells of the four plates (spike antigens, nucleocapsid antigens and positive/negative control); cells were incubated and interferon-γ secreting T cells were detected. Spot-forming units (SFUs) per 250,000 cells were evaluated using an ImmunoSpot S6 ENTRY Analyzer (Cellular Technology Limited, Cleveland, OH USA) and ImmunoSpot 5.0 software (version 7.0.30.2, Cellular Technology Limited). The number of spots from the AIM-V media negative control wells was subtracted from the number of spots in the antigen wells. Ten or more SFU per 250,000 cells in spike antigen plates was determined to be a positive cellular response; the patients whose samples showed positive cellular response were regarded as cellular responders. However, samples that demonstrated ≥ 10 SFU per 250,000 cells in nucleocapsid antigen plates were excluded for the T-SPOT assay because prior SARS-CoV-2 infection or strong non-specific response were suspected, and such samples were not considered appropriate for evaluating vaccine-induced cellular response.

1. Eligible patients for the T-SPOT assay and methodology for further analyses

Among patients included in this study, cryopreserved samples i) obtained at TP1 and TP5 in pairs and ii) with total PBMC>1.5×10^6^ cells, measured before cryoprocessing, were selected and thawed for the T-SPOT assay. The T-SPOT assay was performed using only samples with >1.0×10^6^ cells in total (enough for the analysis of four plates) after thawing.

After excluding one sample at TP1 and two samples at TP5, which showed positive cellular response to SARS-CoV-2 nucleocapsid antigens, 12 samples at TP1 and 12 samples at TP5 were eligible for cellular immune response evaluation, and seven paired samples obtained at TP1 and TP5 were available. The median duration between sample collection and T-SPOT assay implementation was 11 months (interquartile range [IQR], 9.8–11) in TP1 samples and 4 months (IQR, 3–4) in TP5 samples.

**Statistical analysis**

Based on the S-IgG titers at TP1 and TP5, patients were classified as non-responders (≤10 binding antibody units [BAU]/mL), low-responders (>10 and <300 BAU/mL), and adequate-responders (≥300 BAU/mL) to doses 2 and 3. Seroconversion was defined as acquiring an S-IgG titer >10 BAU/mL. The S-IgG titer below the sensitivity (5 BAU/mL) was converted to 1 BAU/mL for statistical analyses.

The clinical parameters associated with being adequate-responders to the second vaccine dose were evaluated using univariate and multivariable analyses. In univariate and multivariable logistic regression analyses, Firth's penalized maximized likelihood method was used to reduce bias in the parameter estimates. The clinical parameters included age (<70 years vs. ≥70 years), sex, vaccine type (BNT165b2 vs. mRNA-1273), anti-myeloma treatment within three months prior to dose 2, and history of autologous stem cell transplantation. The latest data concerning lymphocyte counts in peripheral blood (<1000/µL vs. ≥1000/µL), serum albumin (<3.5 g/dL vs. ≥3.5 g/dL) and estimated glomerular filtration rate (eGFR) (<40/mL/min/1.73 m^2^ vs. ≥40/mL/min/1.73 m^2^), and immunoglobulin (Ig) M levels (<17 mg/dL vs. ≥17 mg/dL) within 60 days prior to the second vaccination were also used. IgG and IgA levels were not used because they included M protein in some patients. The cut-off values for age, lymphocyte count, serum albumin, and eGFR were defined based on clinical significance. The cut-off value for IgM was defined based on the median value for our patients. The clinical information of the patients was reviewed through electronic medical records, up to the data cut-off of Aug 31, 2022. Continuous variables were reported as median (IQR) and compared using the Mann–Whitney U test. Categorical variables were compared using Fisher’s exact test. Multivariable regression analysis was performed to analyze the association between being responders to the second vaccination and various factors. Factors with p value <0.2 in the univariate analysis were included in the multivariable logistic regression analysis with backward stepwise selection.

To compare S-IgG titers between subgroups, the geometric mean titer (GMT) was used. The GMT of S-IgG was calculated as the mean of the assay results after logarithmic transformation and exponentiation of the mean to express results on the original scale. Two-sided 95% confidence intervals were obtained by performing logarithmic transformations of titers or concentrations, calculating the 95% confidence interval with reference to the t-distribution, and then exponentiating the limits of the confidence intervals. The estimated GMTs of samples at TP2 through TP5 were calculated and compared using mixed-effects models for repeated measures to deal with missing data. The GMT between treatment subgroups was compared using t-test.

The results of SFU against SARS-CoV-2 spike antigens were combined with S-IgG titer obtained at the same time point and the correlation between SFU and S-IgG titer was evaluated by Spearman’s correlation analysis. In addition, the SFU in spike antigen plates between the seven paired samples obtained at TP1 and TP5 was compared by paired t-test.

All tests were two-tailed, and p-values less than 0.05 were considered to be statistically significant. Statistical analyses were performed using SAS version 9.4 (SAS Institute, Inc., Cary, NC, USA) and EZR version 1.35 (Saitama Medical Center, Jichii Medical University, Saitama, Japan), which is a graphical user interface for R (The R Foundation for Statistical Computing, Vienna, Austria) (Kanda Y. Bone marrow transplant. 2013; 48: 452-58).
