# Supplementary material for: Humoral and cellular immune response to second and third severe acute respiratory syndrome coronavirus 2 mRNA vaccine in patients with plasma cell dyscrasia
Source: Cancer Med. 2023 Apr 26;12(12):13135–44. doi: 10.1002/cam4.5996 (PMC10315730; doi:10.1002/cam4.5996)
Supplement: Supplementary file 1 — Data S1. [file CAM4-12-13135-s001.zip › CAM4_5996_Table S2_for_revise0130.docx]

| Table S2. Patient information of five low-responders to the third vaccination despite of achieving adequate humoral response to the second vaccination | | | | | | | | | | | | |  |
| --- | --- | --- | --- | --- | --- | --- | --- | --- | --- | --- | --- | --- | --- |
|  |  |  | At the second vaccination | | |  |  |  | At the third vaccination | | |  |  |
| Pt | Age | Sex | mRNA vaccine type | Anti-myeloma treatment | Disease control | S-IgG titer at TP1, BAU/mL | Duration between dose 2 and 3 (months) | Reason of treatment change between dose 2 and 3 | mRNA vaccine type | Anti-myeloma treatment | Disease control | S-IgG titer at TP5, BAU/mL |  |
| 1 | 81 | F | BNT162 | ILd | PR | 2628 | 7.2 | PD | BNT162 | Isa-Pd | PR | 75 |  |
| 2 | 85 | M | BNT162 | Ld | PD | 1928 | 7.8 | PD | BNT162 | D-Ld | VGPR | 258 |  |
| 3 | 66 | M | BNT162 | Off^(a)^ | PR | 4179 | 7.5 | PD | BNT162 | Clinical trial^(b)^ | PR | 44 |  |
| 4 | 57 | M | mRNA1273 | D-Pd | VGPR | 310 | 7.4 | NA | mRNA1273 | D-Pd | PD | 124 |  |
| 5 | 77 | M | BNT162 | ILd | PR | 334 | 6.5 | NA | BNT162 | ILd | PD | 118 |  |
| Pt, patient; TP, time point; TP1, duration defined as within 7–60 days after the second mRNA vaccine dose; TP5, duration defined as within 7 to 60 days after the third mRNA vaccine dose; S-IgG, SARS-CoV-2 antibody against spike proteins; BAU, binding antibody unit; dose 2, second mRNA vaccination; dose 3, third mRNA vaccination; M, male; F, female; I, ixazomib; L, lenalidomide; d, dexamethasone; Isa, isatuximab; P, pomalidomide; D, daratumumab; BCMA, B-cell maturation antigen; PR, partial response; PD, progressive disease; VGPR, very good PR; NA, not applicable (a) The patient received BCMA chimeric antigen receptor T-cell therapy 15 months before the second vaccination. (b) The patient was treated with regimens including B-cell maturation antigen targeted therapy in a clinical trial. | | | | | | | | | | | | | |
