# Supplementary material for: Humoral and cellular immune response to second and third severe acute respiratory syndrome coronavirus 2 mRNA vaccine in patients with plasma cell dyscrasia
Source: Cancer Med. 2023 Apr 26;12(12):13135–44. doi: 10.1002/cam4.5996 (PMC10315730; doi:10.1002/cam4.5996)
Supplement: Supplementary file 1 — Data S1. [file CAM4-12-13135-s001.zip › CAM4_5996_Table S3 for revise.docx]

| Table S3. Univariate analyses for factors associated with being adequate-responders to the third vaccine dose | | | |
| --- | --- | --- | --- |
|  |  | Univariate analysis | |
| Factor | Cut-off | Odds ratio (95% CI) | p |
| Age | < 70 years | - |  |
|  | ≥ 70 years | 0.41 (0.12–1.29) | 0.13 |
| Sex | Male | - |  |
|  | Female | 0.96 (0.32–2.86) | 1.00 |
| Vaccine type | BNT162b2 | - |  |
|  | mRNA-1273 | 3.63 (0.93–21.07) | 0.06 |
| Prior ASCT | No | - |  |
|  | Yes | 2.55 (0.82–8.96) | 0.09 |
| Lymphocyte count in PB | < 1000 /µL | - |  |
|  | ≥ 1000 /µL | 1.40 (0.46–4.23) | 0.61 |
| Serum albumin | < 3.5 g/dL | - |  |
|  | ≥ 3.5 g/dL | 1.10 (0.10–6.86) | 1.00 |
| eGFR | < 40 mL/min/1.73m2 | - |  |
|  | ≥ 40 mL/min/1.73m2 | 1.43 (0.29–5.79) | 0.73 |
| Serum IgM level | < 17 mg/dL | - |  |
|  | ≥ 17 mg/dL | 1.42 (0.44–4.43) | 0.59 |
| CI, confidence interval; ASCT, autologous stem cell transplantation; PB, peripheral blood; eGFR, estimated glomerular filtration rate; Ig, immunoglobulin. | | | |
