# Supplementary material for: Humoral and cellular immune response to second and third severe acute respiratory syndrome coronavirus 2 mRNA vaccine in patients with plasma cell dyscrasia
Source: Cancer Med. 2023 Apr 26;12(12):13135–44. doi: 10.1002/cam4.5996 (PMC10315730; doi:10.1002/cam4.5996)
Supplement: Supplementary file 1 — Data S1. [file CAM4-12-13135-s001.zip › CAM4_5996_Table_S1_clean copy.docx]

| Table S1. Baseline characteristics and treatment information of patients undergoing active anti-myeloma treatment^(a)^ (n = 80) | | | |
| --- | --- | --- | --- |
|  | Non/low-responders | Adequate-responders |  |
|  | n=48 | n=32 | p |
| Anti-myeloma therapy regimen |  |  |  |
| IMiDs, n (%) | 10 (20.8) | 10 (31.2) | 0.017 |
| PI, n (%) | 2 (4.2) | 1 (3.1) |  |
| IMIiDs + PI, n (%) | 4 (8.3) | 8 (25.0) |  |
| Anti-CD38 mAb monotherapy, n (%) | 3 (6.2) | 6 (18.8) |  |
| IMiDs + mAb, n (%) | 14 (29.2) | 3 (9.4) |  |
| PI + anti-CD38 mAb, n (%) | 10 (20.8) | 4 (12.5) |  |
| Novel targeted therapy^(b)^, n (%) | 5 (10.4) | 0 (0.0) |  |
| Regimens including, |  |  |  |
| Anti-CD38 mAb, n (%) | 26 (54.2) | 13 (40.6) | 0.261 |
| IMiDs, n (%) | 30 (62.5) | 21 (65.6) | 0.816 |
| PI, n (%) | 16 (33.3) | 13 (40.6) | 0.636 |
| Prior ASCT, n (%) | 14 (29.2) | 17 (53.1) | 0.038 |
| eGFR < 40 mL/min/1.73m^2^, n (%) | 10 (20.8) | 1 (3.1) | 0.043 |
| Vaccine type, BNT162b2:mRNA-1273, n | 47:1 | 22:10 | < 0.001 |
| Serum IgM level < 17 mg/dL, n (%)* | 14 (29.8) | 22 (68.8) | 0.001 |
| (a) In this study, active treatment was defined as receiving any anti-myeloma treatments within three months before vaccination. (b) Novel targeted therapy includes targeted therapy against B-cell maturation antigen (n = 4) or G protein–coupled receptor, class C group 5 member D (n = 1).  IMiDs, immunomodulatory drugs; PI, proteasome inhibitor; mAb, monoclonal antibody; ASCT, autologous stem cell transplantation; eGFR, estimated glomerular filtration rate; Ig, immunoglobulin.  * As these 80 patients were selected from a population of patients undergoing active anti-myeloma treatment, their median IgM level was different from the median level of the whole patient population, 17 mg/dL. | | | |
