# Supplementary material for: Humoral and cellular immune response to second and third severe acute respiratory syndrome coronavirus 2 mRNA vaccine in patients with plasma cell dyscrasia
Source: Cancer Med. 2023 Apr 26;12(12):13135–44. doi: 10.1002/cam4.5996 (PMC10315730; doi:10.1002/cam4.5996)
Supplement: Supplementary file 1 — Data S1. [file CAM4-12-13135-s001.zip › CAM4_5996_Table_S4_clean copy.docx]

| Table S4. Information of 17 patients eligible for the T-SPOT assay | | | | | | | | | | | | | | | | | |
| --- | --- | --- | --- | --- | --- | --- | --- | --- | --- | --- | --- | --- | --- | --- | --- | --- | --- |
|  |  |  | At the second vaccination | |  | Samples at TP1 | | | |  | At the third vaccination | |  | Samples at TP5 | | | |
| Pt | Sex | Age | Vaccine  type | Regimen^(a)^ |  | S-IgG titre, BAU/mL | SFU to S- antigens | Cellular response decision | SFU to N- antigens | Duration  between  dose 2 and 3  (months) | Vaccine  type | Regimen^(a)^ |  | S-IgG titre, BAU/mL | SFU to  S- antigens | Cellular response decision | SFU to N- antigens |
| 1 | F | 74 | BNT162b2 | D-Ld |  | 540 | 7 | Negative | 3 | 7.6 | BNT162b2 | Off |  | 13390 | NA | NA | NA |
| 2 | M | 71 | BNT162b2 | D-Ld |  | 43 | 4 | Negative | 3 | 7.2 | BNT162b2 | D-Ld |  | 39 | NA | NA | NA |
| 3 | F | 36 | BNT162b2 | E-Ld |  | 57 | 15 | Positive | 0 | 8.4 | BNT162b2 | Clinical Trial^(b)^ |  | 1 | NA | NA | NA |
| 4 | M | 73 | BNT162b2 | Pd |  | 1 | 18 | Positive | 0 | 7.5 | BNT162b2 | Isa-Pd |  | 1 | NA | NA | NA |
| 5 | M | 64 | BNT162b2 | Naive |  | 193 | 30 | Positive | 0 | 8.5 | BNT162b2 | post ASCT |  | 857 | NA | NA | NA |
| 6 | F | 75 | BNT162b2 | D-MPB |  | 135 | NA | NA | NA | 7.1 | BNT162b2 | D-MPB |  | 1707 | 37 | Positive | 1 |
| 7 | F | 70 | mRNA-1273 | Ld |  | 3361 | NA | NA | NA | 7.7 | mRNA-1273 | D-Ld |  | 1802 | 12 | Positive | 0 |
| 8 | F | 71 | BNT162b2 | Off |  | 1834 | NA | NA | NA | 7.6 | mRNA-1273 | D-Ld |  | 3560 | 20 | Positive | 1 |
| 9 | F | 63 | BNT162b2 | D-Ld |  | 90 | NA | NA | NA | 6.2 | BNT162b2 | D-Ld |  | 98 | 4 | Negative | 0 |
| 10 | F | 70 | BNT162b2 | Off |  | 1595 | NA | NA | NA | 7.4 | BNT162b2 | Off |  | 3483 | 14 | Positive | 0 |
| 11 | F | 73 | BNT162b2 | Clinical Trial^(b)^ |  | 1 | 0 | Negative | 0 | 7.8 | BNT162b2 | Clinical Trial^(b)^ |  | 1 | 1 | Negative | 0 |
| 12 | M | 61 | BNT162b2 | Off |  | 2710 | 7 | Negative | 4 | 7.2 | BNT162b2 | Off |  | 8174 | 29 | Positive | 1 |
| 13 | M | 71 | BNT162b2 | D-Ld |  | 90 | 0 | Negative | 0 | 7.3 | BNT162b2 | D-Ld |  | 426 | 8 | Negative | 0 |
| 14 | M | 77 | BNT162b2 | Off |  | 1295 | 1 | Negative | 0 | 6.6 | mRNA-1273 | D-Ld |  | 1038 | 7 | Negative | 0 |
| 15 | F | 79 | BNT162b2 | Off |  | 377 | 15 | Positive | 3 | 8.2 | BNT162b2 | D-Ld |  | 7669 | 44 | Positive | 9 |
| 16 | M | 46 | BNT162b2 | L |  | 432 | 4 | Negative | 0 | 6.4 | mRNA-1273 | L |  | 3160 | 16 | Positive | 0 |
| 17 | F | 62 | BNT162b2 | D |  | 1565 | 99 | Positive | 4 | 7.7 | BNT162b2 | CLd |  | 4022 | 180 | Positive | 0 |

(a) The treatment regimen administrated within 90 days before the vaccination is described. (b) The patients were treated with regimens including B-cell maturation antigen targeted therapy in some clinical trials.

Pt, patient; F, female; M, male; TP, time point; TP1, duration defined as within 7 to 60 days after the second mRNA vaccine dose; TP5, duration defined as within 7–60 days after the third mRNA vaccine dose; S-IgG, SARS-CoV-2 antibody against spike proteins; BAU, binding antibody unit; SFU, spot-forming unit; S-antigens, SARS-CoV-2 spike antigens; N-antigens, SARS-CoV-2 nucleocapsid antigens; dose 2, second mRNA vaccination; dose 3, third mRNA vaccination; NA, not available; D, daratumumab; L, lenalidomide; d, dexamethasone; E, elotuzumab; P, pomalidomide; Isa, isatuximab; ASCT, autologous stem cell transplantation; M, melphalan; P, prednisolone; B, bortezomib; C, carfilzomib; Off, without anti-myeloma treatment within 90 days before the vaccination
